# Supplementary material for: Short- and Long-Term Effectiveness of Low-Level Laser Therapy Combined with Strength Training in Knee Osteoarthritis: A Randomized Placebo-Controlled Trial
Source: J Clin Med. 2022 Jun 15;11(12):3446. doi: 10.3390/jcm11123446 (PMC9225274; doi:10.3390/jcm11123446)
Supplement: Supplementary file 1 [file jcm-11-03446-s001.zip › jcm-1736439-supplementary.pdf]

Journal of
Clinical Medicine

MDPI

Supplementary material for the article entitled Short- and Long-Term Effectiveness of Low-Level Laser Therapy Combined with Strength Training in Knee Osteoarthritis: A Randomized Placebo-Controlled Trial

Outcome data for the individual weeks are prestented in Tables S1-3. Raw reassessment data are prestented in Tables S4-6.

Table S1. Patient-reported outcomes – scores over time with significance levels.

Variables	Week 3	Week 8	Week 26	Week 52
Pain on movement (VAS)				
Laser group	33.12** (*n* = 25)	28.80** (*n* = 25)	31.48** (*n* = 25)	17.08** (*n* = 24)
Placebo group	31.58** (*n* = 24)	31.57** (*n* = 23)	27.23** (*n* = 22)	32.59** (*n* = 22)
Difference between-group	1.54 (-15.86 to 18.94)	-2.77 (-19.41 to 13.88)	4.25 (-16.06 to 24.56)	-15.51 (-29.91 to -1.10)#
Pain at rest (VAS)				
Laser group	15.00 (*n* = 25)	8.68 (*n* = 25)	13.48 (*n* = 25)	8.08 (*n* = 24)
Placebo group	21.42 (*n* = 24)	20.22 (*n* = 23)	16.41* (*n* = 22)	23.32 (*n* = 22)
Difference between-group	-6.42 (-20.26 to 7.42)	-11.54 (-26.07 to 3.00)	-2.93 (-18.71 to 12.85)	-15.24 (-30.78 to 0.31)
Pain at night (VAS)				
Laser group	12.20* (*n* = 25)	12.56** (*n* = 25)	16.32 (*n* = 25)	5.92** (*n* = 24)
Placebo group	20.63** (*n* = 24)	18.22** (*n* = 23)	20.05 (*n* = 22)	22.64 (*n* = 22)
Difference between-group	-8.43 (-22.12 to 5.27)	-5.66 (-19.15 to 7.84)	-3.73 (-23.20 to 15.75)	-16.72 (-31.37 to -2.07)#
Pain globally (KOOS)				
Laser group	63.56** (*n* = 25)	66.00** (*n* = 25)	66.00** (*n* = 25)	69.68** (*n* = 24)
Placebo group	57.64** (*n* = 24)	62.81** (*n* = 23)	59.98** (*n* = 22)	60.23** (*n* = 22)
Difference between-group	5.92 (-3.50 to 15.34)	3.19 (-9.39 to 15.78)	6.02 (-8.38 to 20.43)	9.45 (-4.54 to 23.44)
Disability in ADL (KOOS)				
Laser group	71.23** (*n* = 25)	73.65** (*n* = 25)	71.88** (*n* = 25)	78.06** (*n* = 24)
Placebo group	63.12** (*n* = 24)	68.02** (*n* = 23)	63.96** (*n* = 22)	62.30** (*n* = 22)
Difference between-group	8.11 (-3.51 to 19.74)	5.63 (-7.15 to 18.40)	7.92 (-7.813 to 23.65)	15.76 (1.55 to 29.98)#
Disability in sports/rec. (KOOS)				
Laser group	39.80** (*n* = 25)	40.60** (*n* = 25)	35.20* (*n* = 25)	40.42** (*n* = 24)
Placebo group	31.04 (*n* = 24)	36.74** (*n* = 23)	32.05* (*n* = 22)	31.14 (*n* = 22)
Difference between-group	8.76 (-6.88 to 24.40)	3.86 (-13.13 to 20.85)	3.15 (-14.19 to 20.50)	9.28 (-7.36 to 25.92)
Quality of life (KOOS)				
Laser group	41.76** (*n* = 25)	46.76** (*n* = 25)	44.00** (*n* = 25)	49.21** (*n* = 24)
Placebo group	34.63** (*n* = 24)	40.49** (*n* = 23)	46.02** (*n* = 22)	43.18** (*n* = 22)
Difference between-group	7.13 (-4.15 to 18.42)	6.27 (-6.56 to 19.09)	-2.02 (-16.19 to 12.15)	6.03 (-7.571 to 19.63)
Any analgesic				
Laser group	5 (20.00%)* (*n* = 25)	5 (20.00%)* (*n* = 25)	8 (32.00%) (*n* = 25)	5 (22.73%)* (*n* = 22)
Placebo group	6 (25.00%) (*n* = 24)	5 (20.83%)* (*n* = 24)	8 (38.10%) (*n* = 21)	11 (52.38%) (*n* = 21)
Difference	$p = 0.742$	$p = 1.000$	$p = 0.760$	$p = 0.062$
NSAIDs				
Laser group	0 (0.00%)* (*n* = 24)	1 (4.17%)* (*n* = 24)	2 (8.00%) (*n* = 25)	1 (4.55%)* (*n* = 22)
Placebo group	2 (8.70%) (*n* = 23)	2 (8.70%) (*n* = 23)	3 (14.29%) (*n* = 21)	7 (33.33%) (*n* = 21)
Difference	$p = 0.234$	$p = 0.609$	$p = 0.648$	$p = 0.021$#

Abbrevations: ADL: activities of daily living; KOOS: Knee Osteoarthritis Outcome Scale; NSAIDs: non-steroidal anti-inflammatory drugs; rec.: recreation; VAS: Visual Analogue Scale.

Within-group change from baseline is significant: * $p < 0.05$; ** $p < 0.01$. Between-group difference at a specific week is significant: # $p < 0.05$. The ranges are 95% confidence intervals signifying between-group differences at individual weeks. The global health change data is the median.

Table S2. Physical assessments – scores over time with significance levels.

Variables	Week 3	Week 8	Week 26	Week 52
Knee flexion AROM (degrees)				
Laser group	122.6 (n = 25)	123.6 (n = 25)	124.4* (n = 25)	123.0 (n = 22)
Placebo group	123.8 (n = 24)	124.9 (n = 24)	125.3 (n = 21)	124.4 (n = 21)
Difference between-group	-1.2 (-7.96 to 5.70)	-1.3 (-6.99 to 4.48)	-0.9 (-7.96 to 6.01)	-1.4 (-7.50 to 4.83)
30 seconds chair stands				
Laser group	12.36** (n = 25)	14.28** (n = 25)	15.12** (n = 25)	16.76** (n = 21)
Placebo group	11.67** (n = 24)	13.25** (n = 24)	13.62** (n = 21)	13.10** (n = 21)
Difference between-group	0.69 (-2.28 to 3.67)	1.03 (-2.13 to 4.19)	1.50 (-2.33 to 5.33)	3.67 (-0.29 to 7.63)
Joint line PPT (newton)				
Laser group	45.85 (n = 25)	46.20 (n = 25)	53.29 (n = 25)	49.71 (n = 22)
Placebo group	32.93 (n = 24)	41.97* (n = 24)	44.99** (n = 21)	43.22** (n = 21)
Difference between-group	12.92 (-1.00 to 26.84)	4.23 (-9.69 to 18.14)	8.30 (-9.28 to 25.88)	6.49 (-10.41 to 23.41)
Tibia condyle PPT (newton)				
Laser group	42.25 (n = 25)	44.87 (n = 25)	49.36 (n = 25)	45.91 (n = 22)
Placebo group	31.12 (n = 24)	39.59 (n = 24)	37.77 (n = 21)	38.03 (n = 21)
Difference between-group	11.13 (-2.19 to 24.45)	5.28 (-8.04 to 18.60)	11.59 (-5.318 to 28.49)	7.88 (-8.566 to 24.33)

Abbrevations: AROM: active range of motion; PPT: pain pressure threshold.

Within-group change from baseline is significant: * $p < 0.05$; ** $p < 0.01$. The ranges are 95% confidence intervals signifying between-group differences at individual weeks.

Table S3. Real-time ultrasonography assessments – scores over time with significance levels.

Variables	Week 3	Week 8	Week 26	Week 52
Suprapatellar effusion (mm)				
Laser group	6.303 (n = 24)	5.897 (n = 25)	4.961 (n = 24)	5.929 (n = 23)
Placebo group	4.813 (n = 24)	4.779 (n = 23)	4.237 (n = 21)	4.515 (n = 21)
Difference between-group	1.490 (-0.529 to 3.508)	1.118 (-0.640 to 2.875)	0.724 (-0.924 to 2.371)	1.414 (-0.255 to 3.083)
Meniscal Doppler (mm^2)				
Laser group	2.574 (n = 18)	2.275 (n = 23)	2.139 (n = 13)	1.613 (n = 11)
Placebo group	1.919 (n = 15)	3.454 (n = 18)	3.086 (n = 16)	2.049 (n = 11)
Difference between-group	0.655 (-1.696 to 3.007)	-1.179 (-3.305 to 0.947)	-0.947 (-4.051 to 2.157)	-0.436 (-2.954 to 2.084)
Cartilage thickness (mm)				
Laser group	1.504 (n = 24)	1.507 (n = 25)	1.502 (n = 24)	1.622 (n = 20)
Placebo group	1.434 (n = 23)	1.541 (n = 22)	1.499 (n = 21)	1.465 (n = 21)
Difference between-group	0.070 (-0.191 to 0.33)	-0.034 (-0.322 to 0.253)	0.003 (-0.322 to 0.328)	0.157 (-0.19 to 0.505)

The ranges are 95% confidence intervals signifying between-group differences at individual weeks.

Table S4. Patient-reported outcomes – raw scores over time.

Variables	Week 3	Week 8	Week 26	Week 52
Pain on movement (VAS)				
Laser group	33.12 ± 25.82 (*n* = 25)	28.80 ± 23.35 (*n* = 25)	31.48 ± 29.19 (*n* = 25)	17.08 ± 12.86 (*n* = 24)
Placebo group	31.58 ± 22.27 (*n* = 24)	31.57 ± 22.20 (*n* = 23)	27.23 ± 25.75 (*n* = 22)	32.59 ± 23.15 (*n* = 22)
Pain at rest (VAS)				
Laser group	15.00 ± 19.20 (*n* = 25)	8.68 ± 16.19 (*n* = 25)	13.48 ± 21.02 (*n* = 25)	8.08 ± 14.66 (*n* = 24)
Placebo group	21.42 ± 19.12 (*n* = 24)	20.22 ± 22.53 (*n* = 23)	16.41 ± 21.49 (*n* = 22)	23.32 ± 24.65 (*n* = 22)
Pain at night (VAS)				
Laser group	12.20 ± 15.79 (*n* = 25)	12.56 ± 15.42 (*n* = 25)	16.32 ± 20.76 (*n* = 25)	5.92 ± 10.37 (*n* = 24)
Placebo group	20.63 ± 21.42 (*n* = 24)	18.22 ± 20.68 (*n* = 23)	20.05 ± 29.93 (*n* = 22)	22.64 ± 24.49 (*n* = 22)
Pain globally (KOOS)				
Laser group	63.56 ± 13.50 (*n* = 25)	66.00 ± 17.02 (*n* = 25)	66.00 ± 17.04 (*n* = 25)	69.68 ± 17.08 (*n* = 24)
Placebo group	57.64 ± 12.58 (*n* = 24)	62.81 ± 17.36 (*n* = 23)	59.98 ± 21.16 (*n* = 22)	60.23 ± 19.95 (*n* = 22)
Disability in ADL (KOOS)				
Laser group	71.23 ± 18.22 (*n* = 25)	73.65 ± 16.79 (*n* = 25)	71.88 ± 19.87 (*n* = 25)	78.06 ± 16.61 (*n* = 24)
Placebo group	63.12 ± 13.67 (*n* = 24)	68.02 ± 18.04 (*n* = 23)	63.96 ± 22.27 (*n* = 22)	62.30 ± 20.78 (*n* = 22)
Disability in sports/rec. (KOOS)				
Laser group	39.80 ± 22.91 (*n* = 25)	40.60 ± 24.51 (*n* = 25)	35.2 ± 24.19 (*n* = 25)	40.42 ± 24.53 (*n* = 24)
Placebo group	31.04 ± 20.36 (*n* = 24)	36.74 ± 22.00 (*n* = 23)	32.05 ± 22.70 (*n* = 22)	31.14 ± 19.82 (*n* = 22)
Quality of life (KOOS)				
Laser group	41.76 ± 16.57 (*n* = 25)	46.76 ± 16.22 (*n* = 25)	44.00 ± 18.82 (*n* = 25)	49.21 ± 17.32 (*n* = 24)
Placebo group	34.63 ± 14.64 (*n* = 24)	40.49 ± 18.60 (*n* = 23)	46.02 ± 19.35 (*n* = 22)	43.18 ± 18.84 (*n* = 22)

Abbrevations: ADL: activities of daily living; KOOS: Knee Osteoarthritis Outcome Scale; NSAIDs: non-steroidal anti-inflammatory drugs; rec.: recreation; rec.: recreation; VAS: Visual Analogue Scale.

The variance data is the standard deviation.

Table S5. Physical assessments – raw scores over time.

Variables	Week 3	Week 8	Week 26	Week 52
Knee flexion AROM (degrees)				
Laser group	122.64 ± 9.97 (*n* = 25)	123.60 ± 8.46 (*n* = 25)	124.36 ± 10.65 (*n* = 25)	123.02 ± 8.19 (*n* = 22)
Placebo group	123.77 ± 8.92 (*n* = 24)	124.85 ± 7.39 (*n* = 24)	125.33 ± 7.98 (*n* = 21)	124.36 ± 7.67 (*n* = 21)
30 seconds chair stands				
Laser group	12.36 ± 4.17 (*n* = 25)	14.28 ± 4.86 (*n* = 25)	15.12 ± 5.85 (*n* = 25)	16.76 ± 5.54 (*n* = 21)
Placebo group	11.67 ± 4.08 (*n* = 24)	13.25 ± 3.84 (*n* = 24)	13.62 ± 4.37 (*n* = 21)	13.10 ± 4.43 (*n* = 21)
Joint line PPT (newton)				
Laser group	45.85 ± 21.91 (*n* = 25)	46.20 ± 25.54 (*n* = 25)	53.29 ± 27.74 (*n* = 25)	49.71 ± 24.04 (*n* = 22)
Placebo group	32.93 ± 15.32 (*n* = 24)	41.97 ± 19.68 (*n* = 24)	44.99 ± 18.96 (*n* = 21)	43.22 ± 19.24 (*n* = 21)
Tibia condyle PPT (newton)				
Laser group	42.25 ± 23.09 (*n* = 25)	44.87 ± 20.67 (*n* = 25)	49.36 ± 29.01 (*n* = 25)	45.91 ± 23.90 (*n* = 22)
Placebo group	31.12 ± 12.90 (*n* = 24)	39.59 ± 18.71 (*n* = 24)	37.77 ± 14.55 (*n* = 21)	38.03 ± 18.03 (*n* = 21)

Abbrevations: AROM: active range of motion; PPT: pain pressure threshold.

The variance data is the standard deviation.

Table S6. Real-time ultrasonography assessments – raw scores over time.

Variables	Week 3	Week 8	Week 26	Week 52
Suprapatellar effusion (mm)				
Laser group	6.303 ± 3.184 (*n* = 24)	5.897 ± 2.743 (*n* = 25)	4.961 ± 2.674 (*n* = 24)	5.929 ± 2.216 (*n* = 23)
Placebo group	4.813 ± 2.236 (*n* = 24)	4.779 ± 2.035 (*n* = 23)	4.237 ± 1.577 (*n* = 21)	4.515 ± 2.131 (*n* = 21)
Meniscal Doppler (mm^2)				
Laser group	2.695 ± 3.304 (*n* = 18)	2.317 ± 2.117 (*n* = 23)	2.139 ± 2.115 (*n* = 13)	1.613 ± 1.268 (*n* = 11)
Placebo group	1.860 ± 1.506 (*n* = 15)	3.468 ± 4.463 (*n* = 18)	3.086 ± 4.037 (*n* = 16)	2.049 ± 2.662 (*n* = 11)
Cartilage thickness (mm)				
Laser group	1.504 ± 0.337 (*n* = 24)	1.507 ± 0.395 (*n* = 25)	1.502 ± 0.447 (*n* = 24)	1.622 ± 0.447 (*n* = 20)
Placebo group	1.434 ± 0.365 (*n* = 23)	1.541 ± 0.381 (*n* = 22)	1.499 ± 0.410 (*n* = 21)	1.465 ± 0.421 (*n* = 21)

The variance data is the standard deviation.
